# Cystoscopic hydrodistention characteristics provide clinical and long-term prognostic features of interstitial cystitis after treatment

**DOI:** 10.1038/s41598-020-80252-x

**Published:** 2021-01-11

**Authors:** Wan-Ru Yu, Jia-Fong Jhang, Han-Chen Ho, Yuan-Hong Jiang, Cheng-Ling Lee, Yung-Hsiang Hsu, Hann-Chorng Kuo

**Affiliations:** 1 Department of Nursing, Hualien Tzu Chi Hospital, Buddhist Tzu Chi Medical Foundation, Hualien, Taiwan; 2 Department of Urology, Hualien Tzu Chi Hospital, Buddhist Tzu Chi Medical Foundation and Tzu Chi University, 707, Section 3, Chung-Yang Road, Hualien, Taiwan; 3 Department of Anatomy, Tzu Chi University, Hualien, Taiwan; 4 Department of Pathology, Hualien Tzu Chi Hospital, Buddhist Tzu Chi Medical Foundation and Tzu Chi University, Hualien, Taiwan

**Keywords:** Medical research, Urology

## Abstract

To evaluate the correlations of clinical symptoms, urodynamic parameters, and long-term treatment outcomes with different findings of cystoscopic hydrodistention (HD) in patients with interstitial cystitis/bladder pain syndrome (IC/BPS). This retrospective analysis of 486 patients with IC/BPS investigated baseline clinical symptoms, disease duration, medical comorbidities, urodynamic findings, cystoscopic characteristics [including maximal bladder capacity (MBC) and the presence of glomerulations and Hunner’s lesions], and outcomes according to the five IC/BPS HD subtypes based on the glomerulation grade, MBC, and the presence of Hunner’s lesions. Receiver operation characteristic analysis identified an optimal cutoff value of MBC ≥ 760 ml as a predictor of satisfactory outcomes. Glomerulation grade and MBC were significantly correlated (r =  − 0.403, *P* < 0.001), and both were significantly associated with IC Symptom Index scores. The rate of satisfactory outcomes was better for the patients with low glomerulation grade and MBC ≥ 760 ml (64.2%), and significantly worse for those with Hunner’s lesions (36.8%); no significant differences were noted among the other groups. The results suggested that IC/BPS patients can be classified into the following three distinct subgroups: (1) those with low glomerulation grade and MBC ≥ 760 ml; (2) those with low glomerulation grade and MBC < 760 ml, or with high glomerulation grade regardless of MBC; and (3) those with Hunner’s lesions. The results showed that three IC/BPS subgroups had distinct bladder characteristics and treatment outcomes. The patients with high MBC and low glomerulation grade after HD had more medical comorbidities but a significantly higher rate of satisfactory treatment outcome.

**IRB**: 105-25-B.

## Introduction

Interstitial cystitis/bladder pain syndrome (IC/BPS) is a symptom syndrome characterized by urinary frequency and urgency and bladder pain. Its underlying pathophysiology has not been entirely elucidated, and there is no lasting effective treatment. A high proportion of IC/BPS cases involve the functional somatic syndrome and present with multiple somatic pains and functional disorders^1^. The difficulty with clearly delineating IC/BPS may be due to the heterogeneous clinical presentations and the failure of treatments based on the ambiguous classification of this bladder disorder.

Interstitial cystitis (IC) was first reported based the cystoscopic finding of ulceration in the bladder wall and severe bladder pain at full bladder. Hunner’s lesion was, therefore, considered to be the mainstay finding of IC. Later, it was observed that glomerulations developed after cystoscopic hydrodistention (HD), and the terminology “non-ulcer IC” was proposed^2^. However, the characteristic glomerulations during cystoscopic HD were not observed in several patients suffering from bladder pain, urinary frequency, and nocturia; “bladder pain syndrome” was suggested as a name for this subset of symptoms^3^, and the recent Asian IC guidelines describe it as “hypersensitive bladder syndrome”^4^. The classification of IC/BPS is still under debate, but “non-ulcer IC/BPS” has been widely accepted as the umbrella term for patients who have clinical IC symptoms but without a Hunner’s lesion^3,4^.

According to the definition by the U.S. National Institute of Diabetes and Digestive and Kidney Diseases (NIDDK), IC is a diagnosis of exclusion. Several urinary tract diseases should be excluded before a diagnosis of IC is made^5^. The American Urological Association guidelines suggest IC should be considered for patients who have experienced characteristic symptoms for more than 6 weeks that were refractory to medical treatment^6^. The European Society for the Study of Interstitial Cystitis (ESSIC) has also classified IC/BPS subtypes; the classification is independent of any histopathological findings of chronic inflammation^7^. However, the treatment outcomes for IC/BPS subtypes are inconsistent, suggesting that the classification of IC/BPS based on symptoms might not be suitable for all patients. There is therefore a need to reconsider the classification of IC/BPS based on other clinical characteristics.

Prominent pathological findings from bladder biopsies include urothelial denudation, increased bladder inflammation, and the activation of sensory afferent nerves^8^. An increase in inflammatory cells, focal lymphoid aggregates, and the increased expression of B- and T-cell markers have been observed in IC/BPS bladder tissue^9^. In addition, increased levels of pro-inflammatory proteins have been detected in the serum of patients with IC/BPS, suggesting that the main focus of IC/BPS is indeed the urinary bladder^10^. In clinical practice, careful examination of cystoscopic findings after HD usually found that there was no strict association between the maximal bladder capacity (MBC) and glomerulations grade.

These findings suggest that IC/BPS bladders should be classified into more detailed subtypes than just the presence or absence of Hunner’s lesions, and a possible classification based on cystoscopic findings should be examined to establish whether it can reflect clinical characteristics and treatment outcomes. The aims of this study, therefore, were to formulate a classification of IC/BPS based on findings after cystoscopic HD and to investigate the correlation between the classifications and clinical symptoms, urodynamic parameters, and treatment outcomes.

## Results

**Figure 1:**
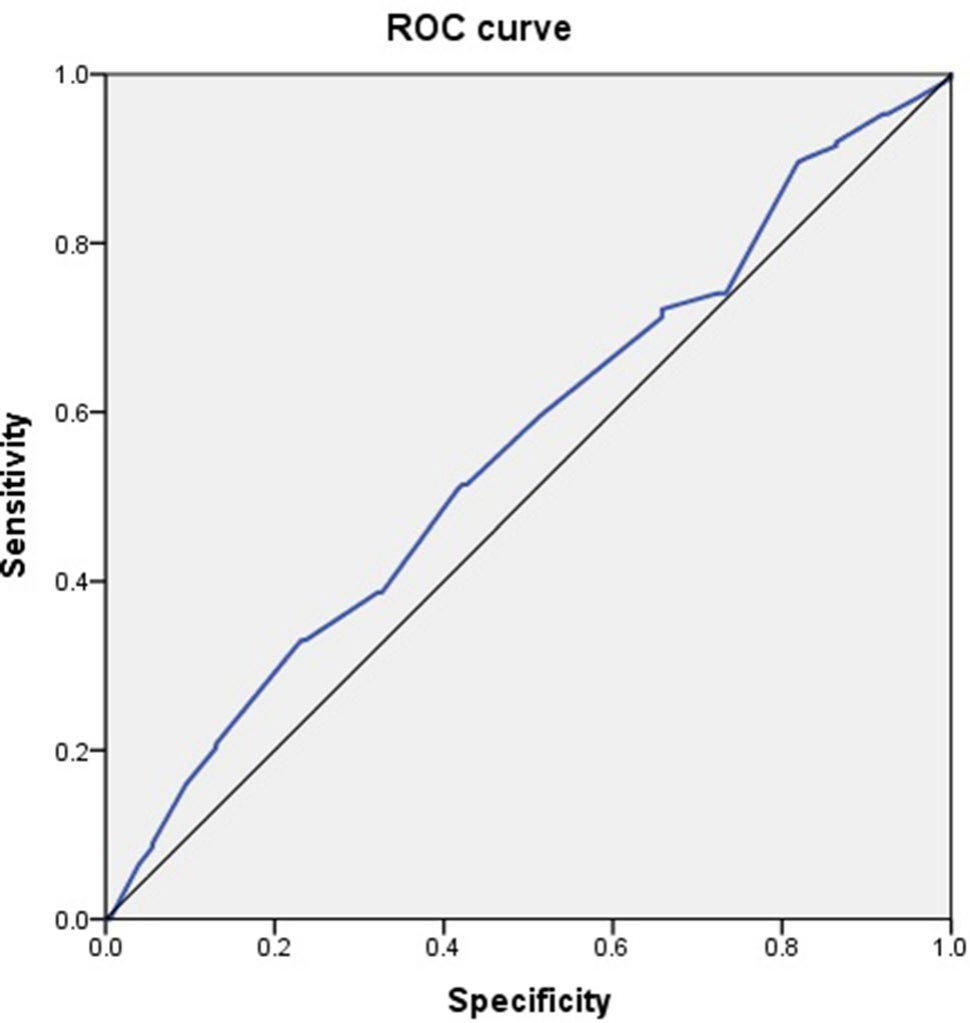
The receiver operation curve of the maximal bladder capacity and global response assessment. With the cut-off value of 760 ml, the area under curve was 0.559, *P* = 0.038.

The receiver operation characteristic analysis showed that the optimal cutoff value for MBC for predicting a satisfactory outcome of global response assessment (GRA) ≥ 2 was ≥ 760 ml. With the cut-off value of 760 ml, the area under curve was 0.559 (*p* = 0.038). (Fig. 1) Together with the glomerulation grade and the presence or absence of Hunner’s lesions, MBC was used to classify the patients into five subgroups: glomerulation grade 0 or 1, MBC ≥ 760 ml (n = 95); glomerulation grade 0 or 1, MBC < 760 ml (n = 107); glomerulation grade 2 or 3, MBC ≥ 760 ml (n = 41); glomerulation grade 2 or 3, MBC < 760 ml (n = 224); and the presence of Hunner’s lesions (n = 19).

Table 1 shows the correlations between clinical symptom scores and cystoscopic HD findings. Glomerulation grade and MBC were significantly inversely correlated (r = − 0.403, *P* < 0.001). Glomerulation was significantly correlated with Interstitial cystitis symptom index (ICSI) and inversely correlated with cystometric bladder capacity (CBC), and MBC was significantly correlated with CBC and inversely correlated with ICSI. Bladder pain measured by the VAS was also significantly associated with ICSI and CBC. Interestingly, the proportion of patients reporting a GRA score ≥ 2 was significantly lower among those with bladder pain than those without bladder pain (95/213, 44.6% vs. 113/191, 59.2%; *P* = 0.009). Similarly, the proportion of patients reporting a GRA score ≥ 2 was significantly lower among those with symptoms of difficult urination than in those without dysuria (39/113, 34.5% vs. 169/292, 57.9%; *P* < 0.001).

**Table 1:** Pearson correlation coefficients for correlations between glomerulation grade or maximal bladder capacity and interstitial cystitis symptom score parameters.

	CBC (ml)	ICSI	ICPI	OSS	VAS
Glomerulation	− 0.251 *P* = 0.000	0.216 *P* = 0.000	0.137 *P* = 0.028	0.189 *P* = 0. 002	0.086 *P* = 0.167
MBC	0.360 *P* = 0.000	− 0.135 *P* = 0.030	− 0.063 *P* = 0.313	− 0.108 *P* = 0.084	0.046 *P* = 0.459

MBC, maximal bladder capacity; CBC, cystometric bladder capacity; ICSI, Interstitial Cystitis Symptom Index; ICPI, Interstitial Cystitis Problem Index; OSS, O’Leary–Sant Symptom score; VAS, visual analog scale score.

Table 2 shows the mean bladder capacities and IC symptom and pain scores according to the different glomerulation grades (or the presence of Hunner’s lesions). As the glomerulation grade increased, the MBC and CBC values significantly decreased. The ICSI, ICPI, OSS, and VAS scores showed no significant difference among the patients of any glomerulation grade, but all except the ICPI scores significantly increased in the patients with Hunner’s lesions.

**Table 2:** Bladder capacity and symptom and pain scores according to the glomerulation grade.

Glomerulation	MBC (ml)	CBC (ml)	ICSI	ICPI	OSS	VAS
0 (n = 26)	743 ± 170	321 ± 144	10.1 ± 4.0	10.6 ± 4.4	20.7 ± 7.9	3.67 ± 2.1
1 (n = 176)	746 ± 168	312 ± 122	11.7 ± 3.8	11.1 ± 3.46	22.8 ± 6.7	4.88 ± 2.4
2 (n = 218)	606 ± 153	279 ± 119	11.9 ± 4.05	11.0 ± 3.93	22.9 ± 7.5	4.31 ± 2.8
3 (n = 47)	593 ± 197	236 ± 87	13.1 ± 3.64	12.1 ± 3.42	25.2 ± 6.8	4.71 ± 2.6
HL (n = 19)	450 ± 138	179 ± 109	16.0 ± 4.33	13.7 ± 3.09	29.7 ± 7.3	6.83 ± 2.9
*P* value	0.000	0.000	0.002	0.099	0.010	0.017
*P* < 0.05	0,1 vs 2,3,4 HL vs all	HL vs 0,1,2	HL vs 0,1,2		HL vs 1,2	HL vs 2

The data are presented as mean ± standard deviation. HL, Hunner’s lesions; MBC, maximal bladder capacity; CBC, cystometric bladder capacity; ICSI, Interstitial Cystitis Symptom Index; ICPI, Interstitial Cystitis Problem Index; OSS, O’Leary–Sant Symptom score; VAS, visual analog scale for pain (0–10).

Higher urothelial dysfunction.

Table 3 compares patient characteristics and IC symptom and pain scores between the five subgroups based on glomerular grade, MBC, and the presence of Hunner’s lesions. The symptoms and pain VAS were similar among the subgroups without Hunner’s lesion, however, the symptom scores were significantly greater in the subgroup of Hunner’s lesion. Interestingly, the proportion of patients reporting a satisfactory treatment outcome (GRA ≥ 2) was significantly higher for the subgroup with glomerulation grade 0 or 1 and MBC ≥ 760 ml (64.2%), and significantly worse for the Hunner’s lesion subgroup (36.8%). There were no significant differences among the other subgroups (40.2 to 43.9%). The results show that symptom scores differed significantly between the IC/BPS patients with and without Hunner’s lesions but not among the non-ulcer subgroups. The number of medical comorbidities in the subgroup of glomerulation grade 0 or 1 and MBC ≥ 760 ml was significantly greater than those for the other subgroups. Table 4 compares the rates of associated autonomic nervous system-related comorbidities between the five subgroups. The subgroup with glomerulation rate 0 or 1 and MBC ≥ 760 ml had the highest rates of arrhythmia and gastroesophageal reflux disease.

**Table 3:** Comparison of characteristics and interstitial cystitis symptom score parameters between the five subgroups of interstitial cystitis/bladder pain syndrome patients.

	Glom 0/1 MBC ≥ 760	Glom 0/1 MBC < 760	Glom 2/3 MBC ≥ 760	Glom 2/3 MBC < 760	Hunner’s Lesion	*P*	*P* < 0.05
Subgroup	1	2	3	4	HL		
N	95	107	41	224	19		
Age (y)	58.9 ± 13.3	61.3 ± 14.0	52.5 ± 13.1	58.3 ± 13.8	66.4 ± 12.6	0.001	HL vs 3, 2 vs 3
IC duration (y)	12.3 ± 7.4	13.4 ± 10.2	12.9 ± 9.9	14.5 ± 10.6	12.8 ± 7.1	0.405	
Male/female	11/84	14/93	5/36	34/190	1/18	0.730	
ICSI	11.8 ± 3.8	11.3 ± 3.9	10.7 ± 4.5	12.4 ± 3.8	16.0 ± 4.3	0.001	HL vs 1, 2, 3
ICPI	11.4 ± 3.3	10.6 ± 3.8	10.0 ± 4.3	11.5 ± 3.7	13.7 ± 3.1	0.033	
OSS	23.3 ± 6.6	21.9 ± 7.0	20.7 ± 8.4	24.0 ± 7.1	30.0 ± 7.3	0.004	HL vs 2, 3
VAS	5.32 ± 2.3	4.13 ± 2.5	4.72 ± 2.6	4.32 ± 2.8	6.83 ± 2.9	0.004	HL vs 2, 4
Comorbidity	2.58 ± 1.6	2.20 ± 1.6	1.91 ± 1.8	1.74 ± 1.7	1.88 ± 1.4	0.006	1 vs 4
Frequent UTI	18 (18.9%)	18 (16.8%)	11 (26.8%)	28 (12.5%)	1 (5.3%)	0.091	
GRA ≥ 2	61 (64.2%)	45 (42.1%)	18 (43.9%)	90 (40.2%)	7 (36.8%)	0.020	

The data are presented as mean ± standard deviation or number (percentage). Glom, glomerulation grade; MBC, maximal bladder capacity (ml); IC, interstitial cystitis; ICSI, Interstitial Cystitis Symptom Index; ICPI, Interstitial Cystitis Problem Index; OSS, O’Leary–Sant Symptom score; VAS, visual analog scale score for pain (0–10); GRA, Global Response Assessment score (GRA ≥ 2 indicates a satisfactory outcome); UTI, urinary tract infection.

**Table 4:** Frequencies of various autonomic nervous system-related comorbidities, compared between the five patient subgroups.

	Glom 0/1 MBC ≥ 760	Glom 0/1 MBC < 760	Glom 2/3 MBC ≥ 760	Glom 2/3 MBC < 760	Hunner’s Lesion	*P*
N	95	107	41	224	19	
Depression	21 (22.1%)	24 (22.4%)	4 (9.8%)	45 (20.1%)	5 (26.3%)	0.444
Insomnia	37 (38.9%)	46 (43.0%)	10 (24.4%)	75 (33.5%)	9 (47.4%)	0.156
Irritable bowel disease	4 (4.2%)	5 (4.7%)	6 (14.6%)	10 (4.5%)	0 (0.0%)	0.060
Arrythmia	15 (15.8%)	7 (6.5%)	6 (14.6%)	12 (5.4%)	2 (10.5%)	0.019
Gastroesophageal reflux disease	38 (40.0%)	33 (30.8%)	14 (34.1%)	53 (23.7%)	5 (26.3%)	0.050
Asthma	6 (6.3%)	6 (5.6%)	2 (4.9%)	5 (2.2%)	0 (0.0%)	0.304

The data are presented as number (percentage). Glom, glomerulation grade; MBC, maximal bladder capacity (ml).

Table 5 compares the urodynamic parameters between the five subgroups. The results for the middle three groups were similar and so could be combined into a single group. Thus, the patients would be divided into three subgroups: (1) patients with glomerulation grade 0 or 1 and MBC ≥ 760 ml; (2) patients without a Hunner’s lesion but with glomerulation grade 0 or 1 and MBC < 760 ml, or glomerulation grade 2 or 3, regardless of the MBC; and (3) patients with Hunner’s lesions. Based on the urodynamic and cystoscopic HD results, it seems rational to subgroup IC/BPS patients into three subgroups, as follows: hypersensitive bladder (glomerulation grade 0 or 1, MBC ≥ 760 ml); non-ulcer IC (including patients without Hunner’s lesions but with glomerulation grade 0 or 1 and MBC < 760 ml, or with glomerulation grade 2 or 3, regardless of the MBC); and ulcer-type IC (with Hunner’s lesions).

**Table 5:** Comparison of urodynamic parameters between the five subgroups.

	Glom 0/1 MBC ≥ 760	Glom 0/1 MBC < 760	Glom 2/3 MBC ≥ 760	Glom 2/3 MBC < 760	Hunner’s lesion	*P*	*P* < 0.05
Subgroup	1	2	3	4	HL		
N	95	107	41	224	19		
FSF (ml)	141 ± 54	118 ± 47	119 ± 57	113 ± 49	82.4 ± 45	0.000	1 vs 2, 4, HL
FS (ml)	219 ± 71	182 ± 67	198 ± 75	181 ± 66	117 ± 62	0.000	HL vs 1,2,3,4 1 vs 2,4, HL
US (ml)	264 ± 82	225 ± 76	253 ± 95	226 ± 79	132 ± 67	0.000	HL vs 1,2,3,4 1 vs 2,4, HL
CBC (ml)	349 ± 129	281 ± 112	304 ± 120	266 ± 113	179 ± 109	0.000	HL vs 1,2,3,4 1 vs 2,4, HL
Pdet (cmH_2_O)	23.1 ± 12.8	21.9 ± 14.5	21.0 ± 11.7	22.6 ± 27.9	16.3 ± 11.6	0.762	
Qmax (ml/s)	14.2 ± 6.7	11.8 ± 6.5	13.6 ± 5.5	12.6 ± 10.0	9.9 ± 4.3	0.102	
Volume (ml)	322 ± 14	241 ± 106	298 ± 116	232 ± 102	129 ± 88	0.000	HL vs 1,2,3,4 1 vs 2,4, HL
PVR (ml)	26.3 ± 51	40.4 ± 97.8	12.7 ± 21.8	33.6 ± 66.1	49.3 ± 96.1	0.175	
Compliance	87.6 ± 82	70.7 ± 72	63.8 ± 66	58.8 ± 60	34.1 ± 26	0.002	1 vs 4, HL
UDS DV	9 (9.5%)	10 (9.3%)	4 (0.8%)	18 (8.0%)	1 (5.3%)	0.964	
BND	4 (4.2%)	6 (5.6%)	1 (2.4%)	7 (3.1%)	0 (0%)	0.684	
PRES	40 (42.1%)	48 (44.9%)	15 (36.6%)	111 (49.6%)	10 (52.6%)	0.467	
HSB	68 (71.6%)	90 (84.1%)	31 (75.6%)	210 (93.8%)	19 (100%)	0.000	
DO	11 (11.6%)	14 (13.1%)	4 (9.8%)	24 (10.7%)	4 (21.1%)	0.706	

The data are presented as mean ± standard deviation or number (percentage). Glom, glomerulation grade; MBC, maximal bladder capacity (ml); FSF, first sensation of filling; FS, full sensation; US, urge sensation; CBC, cystometric bladder capacity; Pdet, voiding detrusor pressure; Qmax, maximum flow rate; PVR, post-void residual urine; UDS, urodynamic study; DV, dysfunctional voiding; BND, bladder neck dysfunction; PRES, poor relaxation of external sphincter; HSB, hypersensitive bladder; DO, detrusor overactivity.

## Discussion

This study demonstrated that patients with IC/BPS could be stratified according to the findings of cystoscopic HD. The clinical characteristics, symptoms, urodynamic parameters, and findings of cystoscopic HD are distinct between patients with Hunner’s lesions and those with non-ulcer IC/BPS. Among the four non-ulcer IC/BPS subgroups in this study, the patients with glomerulation grade 0 or 1 and MBC ≥ 760 ml exhibited better urodynamic parameters and the highest rate of satisfactory treatment outcome. Interestingly, this subgroup of patients also had significantly more medical comorbidities than the other subgroups.

The most acceptable classification of IC/BPS has been a division between ulcer and non-ulcer subtypes, which have distinct clinical characteristics, cystoscopic findings, and treatment options^11,12^. These two subtypes exhibit a marked, significant difference in bladder capacity under general anesthesia^13^. Although bladder or pelvic pain is considered the cardinal symptom of IC/BPS, patients with mild or moderate IC/BPS symptoms sometimes present with lower abdominal discomfort, and urinary frequency and urgency, but reporting no pain^6,7^. A diagnosis of hypersensitive bladder has been suggested for patients with IC symptoms but no obvious bladder pain^4^.

Many studies have discussed the clinical phenotype of IC/BPS. The UPOINTS phenotyping system was proposed for classifying IC/BPS according to urinary, psychosocial, organ-specific, infection, neurological/systemic, tenderness, and sexual domains^14^. This system helps clinicians to identify the somatic and psychological impact of IC/BPS on the clinical presentation. A study reported that, compared to controls, patients with IC/BPS showed significant cognitive and psychosocial changes^15^. The same group also reported that irritable bowel syndrome, fibromyalgia, and chronic fatigue syndrome were more prevalent in IC/BPS patients than in controls^16^. In addition, the same study reported that greater systemic pain symptoms outside the bladder region were associated with poorer patient outcomes. These findings all suggest that IC/BPS might be one eruption site of the systemic involvement of inflammatory disease.

It is been reported that cystoscopic findings of IC/BPS after HD were not significantly correlated with the degree of bladder inflammation^17^. However, previous studies have demonstrated a lower bladder capacity under anesthesia is associated with greater IC symptoms and significantly more acute and chronic inflammation^18^. In the present study, we found that MBC was significantly associated with glomerulation grade in patients with IC/BPS. When we stratified patients according to a combination of MBC and glomerulation grade, the urodynamic parameters showed a progression from low glomerulation grade and high MBC to high glomerulation grade and low MBC in the patients with non-ulcer type IC/BPS. The treatment outcome was distinct among three subgroups: (1) patients with glomerulation grade 0 or 1 and MBC ≥ 760 ml; (2) patients with non-ulcer IC, including those with glomerulation grade 0 or 1 and MBC < 760 ml, or with glomerulation grade 2 or 3 regardless of the MBC; and (3) ulcer-type IC (with Hunner’s lesions).

It has been reported that patients with IC/BPS are at higher risk of comorbid autoimmune or neurological diseases^1,19,20^. A distinct phenotype of IC/BPS patients with multiple psychosomatic sensitivities has been identified^21^. Crosstalk in pelvic organs has been suggested as the possible pathophysiology for a specific IC/BPS subtype in which patients usually present with pelvic organ pain, irritable bowel syndrome, or myofascial pain in addition to the cardinal bladder symptoms^22^. Patients with a higher bladder capacity are likely to report depression and irritable bowel syndrome^23^. In the present cohort, the patients with glomerulation grade 0 or 1 and MBC ≥ 760 ml had a higher number of comorbidities, which might be a reflection of this proposed pathophysiology.

Recent study suggested some inflammatory mediators, such as in systemic inflammatory diseases, might play crucial roles in the pathogenesis of IC/BPS^10^. IC/BPS patients with low anesthetic bladder capacity had histological evidence of significantly more acute and chronic inflammation, supporting that the low bladder capacity might be associated with a distinct bladder-centric IC/BPS phenotype^18^. These clinical and proteomic results imply that IC/BPS might involve not only the disorders confined to the bladder but also caused or aggravated by a mental factor such as internal conflict or stress disorders^24^. The patients’ bladder symptoms might, in part, result from the effects of systemic medical comorbidities.

Based on the results of this study, it is rationale to classified IC/BPS as different subtypes according to the cystoscopic HD characteristics of MBC and glomerualtion or Hunner’s lesion. Patients with grade 0/1 glomerulation and MBC ≥ 760 ml represent a mild IC subtype and little bladder disease. Patients with Hunner’s lesion have the worst bladder condition and clinical symptoms, and worst treatment results. The other IC subtypes might have higher grade of urothelial dysfunction (glomerulation 2/3) or with higher grade of bladder wall inflammation (small MBC), and have less favorable treatment outcome.

## Materials and methods

### Patients

This study retrospectively analyzed the data for a large cohort of patients with IC/BPS. The study included patients who attended our hospital between October, 1997 and March, 2019 with clinical symptoms of urinary frequency, urgency, and nocturia, with or without bladder pain. Patients had been treated with lifestyle modification and medication for the bladder symptoms such as antimuscarinics, beta-3 adrenoceptor agonists, and non-steroid anti-inflammatory agents (NSAID) but the bladder symptoms remained. Patients with chronic urinary retention, acute or chronic urinary tract infection, urodynamic stress incontinence, pelvic organ prolapse, possible neurogenic voiding dysfunction, previous genital tract or lower urinary tract surgery, previous irradiation, or a previous history of genitourinary tract malignancy were excluded from the analysis. All the patients underwent a videourodynamic study (VUDS) and cystoscopic HD examination. Based on the VUDS results, patients with intrinsic sphincter deficiency, neurogenic bladder, or overt bladder outlet obstruction were excluded from the analysis.

Finally, 486 patients were diagnosed to have IC/BPS and included in this study (65 men, 421 women). Data for their baseline clinical symptoms, disease duration, medical co-morbidity, urodynamic findings, and cystoscopic characteristics (MBC, glomerulations, and Hunner’s lesion) were extracted from the medical records. This included scores for the ICSI, Interstitial Cystitis Problem Index (ICPI), and O’Leary–Sant Symptom index (OSS)^25^.

The study was approved by the Research Ethics Committee of the Hualien Tzu Chi Hospital (IRB: 105-25-B). Because of the retrospective nature of the study, the requirement for informed consent was waived. All methods used in this study were carried out in accordance with relevant guidelines and regulations.

### Videourodynamic study

Prior to any treatment, the patient underwent a VUDS using a multi-channel urodynamic system and a C-arm fluoroscope. With the patient positioned in a supine position, a 6-Fr dual-channel urethral catheter was inserted transurethrally to record the intravesical pressure and an 8-Fr rectal balloon catheter was used to record the intra-abdominal pressure. The VUDS was performed by the infusion of warmed normal saline containing 20% Urografin contrast medium at a filling rate of 20–30 ml/min, with the patient in a sitting position. The fluoroscope was positioned 45 degrees from the buttocks so that the urethra could be lengthened and the bladder neck, urethral sphincer, and distal urethra could be clearly identified. The study was repeated if abnormal findings were noted during the first examination.

The urodynamic variables measured included the CBC, voiding detrusor pressure, maximum flow rate, post-void residual urine, and bladder compliance. A voiding detrusor pressure greater than 35 cm H_2_O was defined as high voiding pressure^26^. Bladder compliance at bladder capacity was measured by dividing the change in cystometric volume by the change in detrusor pressure. Detrusor overactivity was defined according to the recommendation of the International Continence Society^27^. Pain sensation was graded based on a 10-point visual analog scale (VAS).

A final diagnosis of lower urinary tract dysfunction was made based on the main VUDS findings and clinical characteristics. Patients with increased bladder sensation, with or without a positive potassium chloride sensitivity test result, were encouraged to undergo cystoscopic HD.

### Cystoscopic hydrodistension

**Figure 2:**
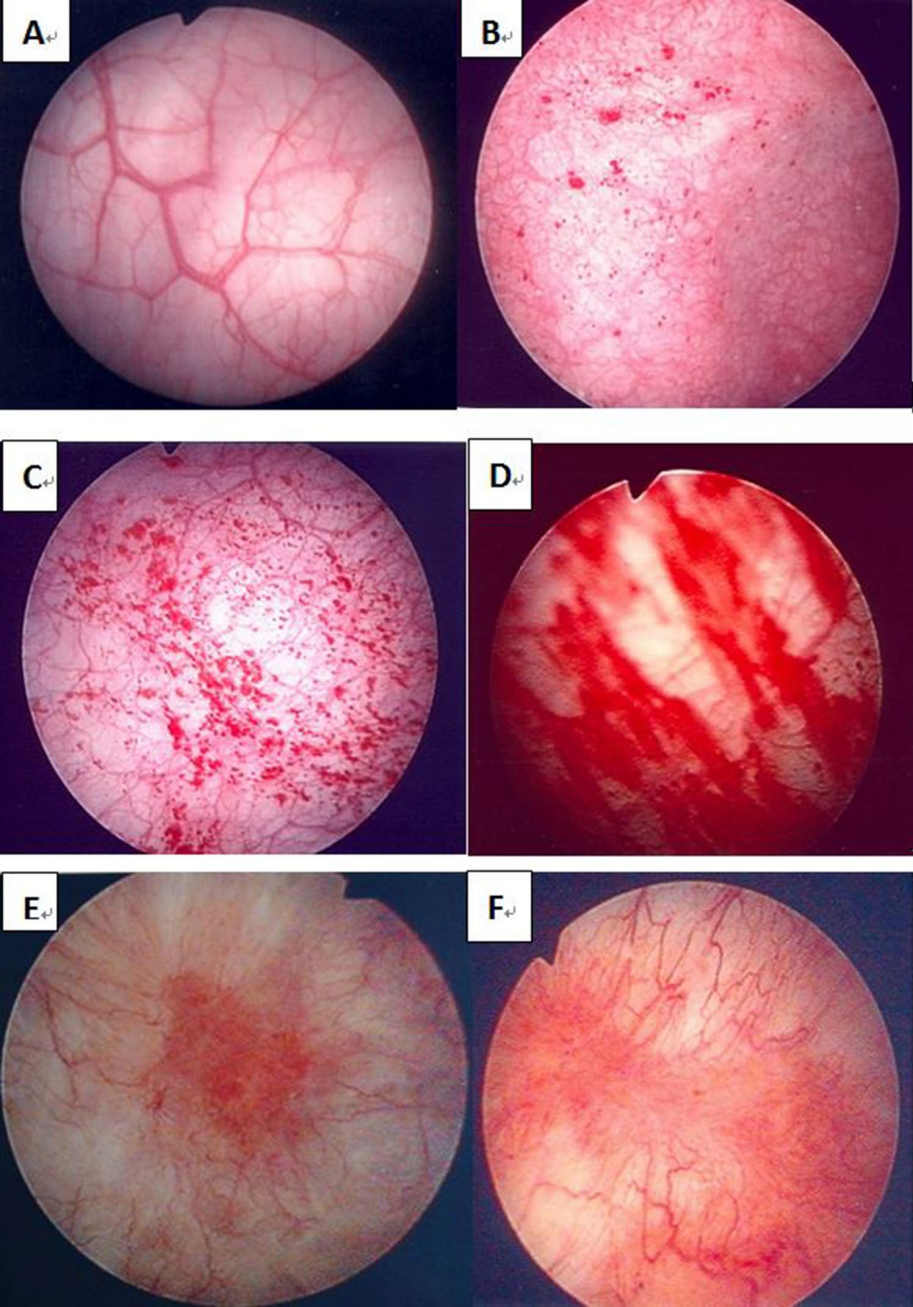
The pictures of cystoscopic hydrodistention of interstitial cystitis after hydrodistention: (**A**) grade 0, (**B**) grade 1, (**C**) grade 2, (**D**) grade 3, (**E**,**F**) Hunner’s lesion.

The patients were admitted for cystoscopic HD, which was performed under general anesthesia. HD was performed under an intravesical pressure of 80 cm H_2_O for 10 min and the bladder was then evacuated slowly. The bladder was carefully examined for the formation of petechia, glomerulations, splotch hemorrhage, mucosal fissures, or ulceration^4^. The glomerulation grade was classified according to the appearance of glomerulations as follows: 0, none; 1, less than half of the bladder wall; 2, more than half of the bladder wall; or 3, severe waterfall bleeding^4^. Patients with Hunner’s lesions with or without glomerulation were classified as having ulcer-type IC/BPS. (Fig. 2) The results of the cystoscopy and bladder HD were obtained from the operative report in the patient’s chart.

### Treatment and outcome assessment

After the cystoscopic HD, the IC patients were consecutively treated with bladder targeting medications for bladder pain such as NSAID, cox-2 inhibitor and antimuscarinics, intravesical hyaluronic acid instillations, or intravesical botulinum toxin A injections and were regularly followed up at outpatient clinic. Most of the patients had received at least three kinds of treatment including hyaluronic acid instillation, anti-inflammatory medication, pain killer drugs, or botulinum toxin A injection. Patients with Hunner’s lesion were further treated with electrocauterization, laser ablation, partial cystectomy with or without augmentation enterocystoplasty according to their treatment outcome to the initial treatment. If they were responding to any kind of treatment, they would not proceed to the next line therapy. The treatment outcome was examined after long-term follow-up according to the patients’ self-reported Global Response Assessment (GRA), scored from -3 (markedly worse) to + 3 (markedly improved)^28^ GRA scores of + 2 or + 3 were considered to represent a satisfactory treatment outcome; all other scores were considered an unsatisfactory outcome. Receiver operation characteristic analysis was used to define the optimal cutoff value of MBC that predicted a satisfactory outcome (i.e., GRA ≥ 2). The patients were classified into subgroups according to their glomerulation grade, MBC, or combined glomerulation grade and MBC.

### Statistical analysis

The patients’ clinical symptoms, clinical presentations, urodynamic findings, and treatment outcomes were analyzed according to the different IC/BPS subtypes. Differences in numerical variables were evaluated with the independent t test and differences in categorical variables with Pearson’s chi-square test. Pearson’s correlation analysis was used to measure the degree of association between pairs of continuous variables and ANOVA was used for analysis the difference among subgroups. Receiver operation characteristic analysis was defined by the Youden index. SPSS 12.0 (SPSS Inc., Chicago, IL, USA) was used for the analyses, and *P* < 0.05 was considered to indicate statistical significance.

## Conclusions

The results showed that patients with IC/BPS can be classified into three subgroups: (1) patients had glomerulation grade 0 or 1 and MBC ≥ 760 ml; (2) non- Hunner’s IC, patients had glomerulation grade 0 or 1 and MBC < 760 ml, or with glomerulation grade 2 or 3 regardless of the MBC; (3) patients with Hunner’s lesions. The patients with a large MBC and low glomerulation grade after HD had a greater number of medical comorbidities but experienced significantly better treatment outcomes.
